# Genetic diversity of *Bacillus anthracis* Ames lineage strains in China

**DOI:** 10.1186/s12879-020-4867-5

**Published:** 2020-02-14

**Authors:** Enmin Zhang, Huijuan Zhang, Jinrong He, Wei Li, Jianchun Wei

**Affiliations:** 1 National Institute for Communicable Disease Control and Prevention, Chinese Center for Disease Control and Prevention, Beijing, China; 2 State Key Laboratory of Infectious Disease Prevention and Control, Beijing, China; 3 Collaborative Innovation Center for Diagnosis and Treatment of Infectious Disease, Hangzhou, China

**Keywords:** Anthrax, *Bacillus anthracis*, Ames lineage strains, canSNP, MLVA

## Abstract

**Background:**

Anthrax is an endemic disease that persists in the rural regions of China. The global genetic population structure of *B.anthracis* has also been defined by the canonical single-nucleotide polymorphisms (canSNP) and multiple-locus variable-number tandem repeat analysis (MLVA). Five canSNP lineages were found in China, and the A.Br.Ames lineage has been the second predominant group in recent years in China. The objective of this study was to reveal genetic diversity of the Ames lineage strains by MLVA.

**Methods:**

Two molecular typing methods, canSNP and MLVA with 15markers were used to study the genetic relationship among the Ames lineage strains. The outbreak information associated with these strains was also collected and investigated.

**Results:**

From 2007 to 2018, a total of 21 human anthrax infection outbreaks (68 patients) associated with *B. anthracis* Ames lineage strains were reported in China. Ames lineage strain-associated human anthrax is mainly distributed in the northern part of China, including the provinces of Inner Mongolia, Liaoning, Gansu, and Xinjiang. In the study, a total of 30 Ames lineage strains were included and 10 MLVA15 genotypes were identified. These strains were mainly found in northeast China, Inner Mongolia and Liaoning. In recent years, the Ames lineage strains were isolated in the two provinces every year. The 18 Ames lineage strains isolated from Inner Mongolia were divided into eight MLVA15 genotypes. From 2010 to 2015, there were continuous reports of outbreaks in Keyouzhongqi County, Inner Mongolia, and the strains that were isolated annually in succession belonged to the MLVA15–30 genotype.

**Conclusions:**

The Ames lineage strains are widely distributed in northern China. Their genetic diversity can be illustrated by the results of the MLVA. The genetic characteristics of the Ames lineage strains from outbreaks in different provinces varied. In some areas, human anthrax outbreaks occurred annually in succession, and these related strains grouped together. These observations indicate that the local environment was persistently contaminated with *B. anthracis* spores, vaccination of livestock should become the fundamental control measure in the areas.

## Background

Anthrax is an acute zoonotic infectious disease caused by *Bacillus anthracis*. *B. anthracis* forms dormant spores that are resistant to extreme environmental conditions and persist in soil for long periods. Herbivorous animals, such as cattle, sheep, and goats, ingest spores in the soil and become infected. There are three primary forms of the disease in humans, including inhalational, gastrointestinal, and cutaneous anthrax. Inhalational anthrax is caused by inhaling aerosolized *B. anthracis* spores*. B. anthracis* has long been considered a potential biological weapon. In the fall of 2001, *B. anthracis* spores were spread through letters mailed in the United States, resulting in 22 cases of anthrax and 5 deaths [1]. These anthrax bioterrorism attacks affirmed the potential of *B. anthracis* as a biological weapon, and the strains were found to belong to the *B. anthracis* A.Br.Ames lineage [2].

Investigations of bioterrorism-associated anthrax led to the establishment and improvement of a molecular typing method. *B. anthracis* is a relatively homogeneous bacterial species [3] due to its long lifecycle, which includes dormant endospores. The global phylogenetic structure of *B. anthracis* was previously defined using a canonical single-nucleotide polymorphisms (canSNPs) method [4] in which 13 representative SNPs were used to establish a SNP-derived phylogenetic tree. The global genetic population polymorphisms were previously defined using multiple-locus variable-number tandem repeat analysis (MLVA) with 8, 15, 25, and 31 markers [5–8]. These strategies were used to trace the sources of naturally occurring anthrax outbreaks. In the worldwide population of *B. anthracis*, three major lineages (A, B and C) and 12 minor lineages/groups were identified; five canSNP lineages/groups were found in China, including A.Br.001/002, A.Br.Ames, A.Br.Vollum, A.Br.Aust94, and A.Br.008/009. The A.Br.Ames lineage descended from the A.Br.001/002 lineage, which has a major presence in China [9]. The phylogenetic analysis also indicated that the original Ames strain and a subset of 10 Texas Ames-like isolates as well as Ames-like isolates from China shared common ancestors that originated in Inner Mongolia, China [9]. In the USA, the Ames strains were thought to be rare in nature even though other Ames isolates were shown to be closely related to the Ames strain isolated in Texas [10]. A number of *B.anthracis* strains were collected in Chinese CDC, these strains had been checked using 13 canSNP markers according to Van Ert [4] and assigned to different canSNP lineages. Strains belonging to the Ames lineage were isolated in China as early as 1954 [11]. During 2007–2018, a total of 99 strains (Liaoning, Sichuan, Yunnan, Guizhou, Shaanxi, Gansu, Beijing, Xinjiang and Inner Mongolia) were collected in Chinese CDC, 71 were assigned to lineage A.Br.001/002, 27 to A.Br.Ames and 1 to A.Br.008/009 (National surveillance data). The Ames lineage has been the second predominant group in recent years. In fact, human anthrax outbreaks caused by the Ames lineage strains are frequently reported in the northern regions of China [11, 12]. In this study, we reported 21 outbreaks associated with Ames lineage strains from 2007 to 2018 in China. To reveal genetic diversity of these isolates and to investigate the potential relationships of strains responsible for persistent human anthrax outbreaks, a total of 30 Ames lineage strains were further identified by MLVA.

## Methods

### Case definition

Human anthrax cases, including probable and confirmed cases, were diagnosed according to the unified case definition issued by the Chinese Ministry of Health in 2008. A probable case was defined as an individual patient who had clinical manifestations with an epidemiological history as well as the demonstration of *B. anthracis* in a clinical specimen through the microscopic examination of a stained smear. A confirmed case was defined as a case with clinical manifestations and the isolation of *B. anthracis* or a ≥ 4-fold increase in specific antibody titers against *B. anthracis.* Human anthrax infection associated with *B. anthracis* A.Br.Ames strains was defined according to the isolation of *B. anthracis* A.Br.Ames or by direct epidemiological links to these strains.

### Strains and DNA preparation

A total of 30 Ames lineage strains were used in this study. These strains were isolated between 1954 and 2018 in the provinces of Inner Mongolia, Xinjiang, Liaoning, Guangxi and Gansu. All strains were collected from the National Institute for Communicable Disease Control and Prevention at the Chinese CDC. *B. anthracis* strains were streaked onto LB agar plates and incubated at 37 °C for 16–18 h. Single colonies were suspended in 0.5 ml TE buffer (10.0 mM Tris-HCl [pH 8.0] and 1.0 mM EDTA) and incubated at 100 °C for 10 min. Next, cellular debris was removed by centrifugation at 15,000×g for 10 min, and the supernatant was collected and filtered using a 0.22-μm filter. The filtered supernatant was diluted 1:10 with sterile nuclease-free H_2_O and was used as DNA template for PCR amplification. Bacterial culturing and DNA preparation were performed in a Bio-Safety level 3 (BSL-3) laboratory.

### MLVA genotyping

We used MLVA with 15 markers, including eight initially described by Keim et al. and seven by Van Ert et al. [4]. The MLVA15 analysis was performed as described previously [4, 12]. Brief description is as follows. The forward primers were labeled with the fluorescent dyes Fam or Hex. PCR amplifications were performed on a SensoQuest Labcycler (SensoQuest, Germany). The PCR products were analyzed by capillary electrophoresis on an ABI 3730xl genetic analyzer using a GeneScan 1200 LIZ size standard (Applied Biosystems). The lengths of the PCR products were determined according to their sizes using GeneMapper software V. 4.0 (Applied Biosystems). Selected PCR products were sequenced to verify the tandem repeat sequences. The electrophoretic band sizes in this study were corrected according to the sequencing results of the PCR products.

### Cluster analysis of the MLVA data

Data were imported as character data sets using the BioNumerics software package (version 5.10, Applied-Maths). In addition to the VNTR data for our 30 A.Br.Ames isolates, additional MLVA15 typing datasets for 11 A.Br.Ames strains from previous studies were also included in the clustering analysis. The MLVA profiles were processed by clustering analysis using the categorical coefficient and the unweighted pair-group method with arithmetic means. Cluster analyses of the categorical data are presented in dendrogram.

## Results

### Anthrax outbreaks associated with Ames lineage strains in China

**Fig. 1:**
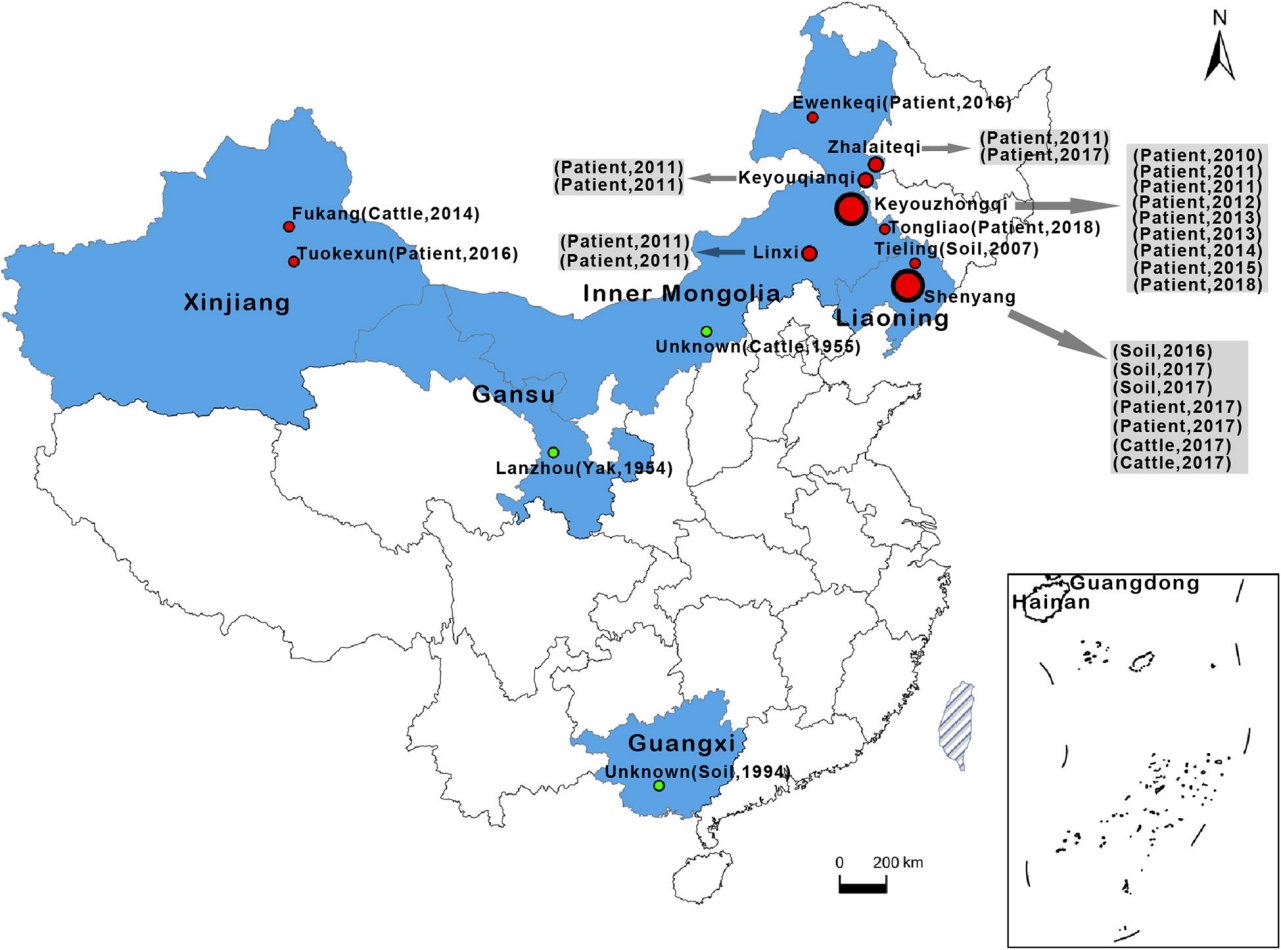
Geographical distribution of *B. anthracis* Ames lineage strains in China. The blue areas indicate the provinces with Ames lineage strains outbreaks. Red and green dots indicate the counties where bacterial cultures were isolated and analyzed. The red dots represent the strains isolated from 2007 to 2018, and the green dots represent the strains isolated before 2007. The sources and year of isolation are listed in brackets. We used ArcGIS version 10.0 (ESRI, USA) and Photoshop CS 8.0.1 (Adobe Systems Incorporated, USA) to plot the map

When the Ames lineage strains were identified, the associated outbreak information was recalled and analyzed. A total of 21 human anthrax outbreaks (68 patients) occurred in China from 2007 to 2018 that were associated with *B. anthracis* Ames lineage strains. The anthrax outbreaks were distributed in the provinces of Inner Mongolia, Liaoning, and Xinjiang (Autonomous Region) (Fig. 1). The Ames lineage strains were isolated from Inner Mongolia in as early as 1955, and they are primarily distributed in the northern regions of China. Guangxi (1994) is the only southern province that reported Ames lineage strain-related infections. In addition, based on their isolation frequencies, more Ames lineage strains were found in Inner Mongolia (17 of 27, 2007–2018), where six counties suffered human infections: Keyouzhongqi, Keyouqianqi, Zhalaiteqi, Linxi, Ewenkeqi and Tongliao. Of these outbreaks, Keyouzhongqi County had the highest number of infections, and human anthrax outbreaks associated with the Ames lineage strains were continually reported from 2010 to 2015. All patients were infected through contact with diseased livestock or contaminated animal products. These observations indicate that the local environment was persistently contaminated with *B. anthracis* spores.

### MLVA and the genetic characteristics of the *B. anthracis* Ames lineage strains

**Fig. 2:**
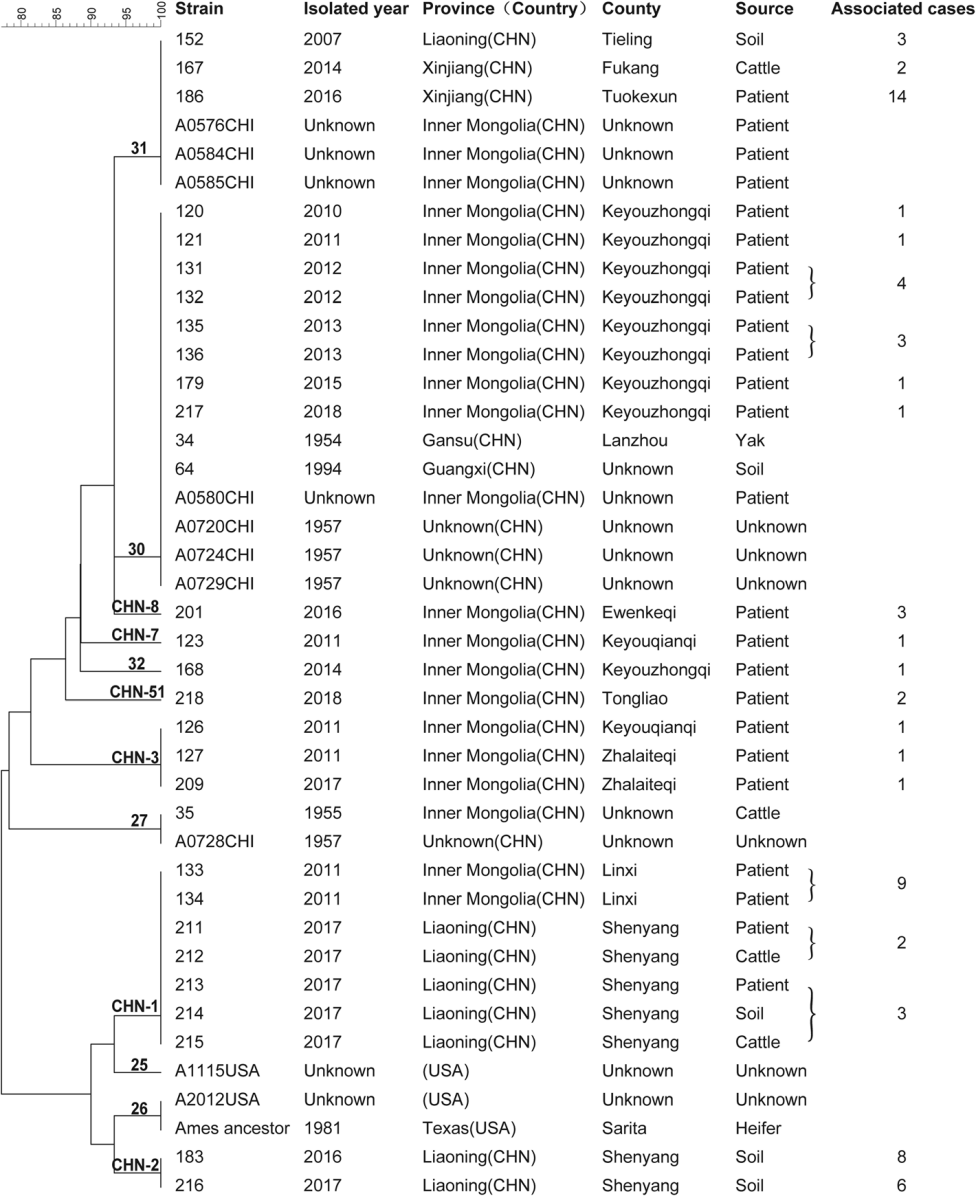
Dendrogram of the genetic relationships of the MLVA genotypes. The MLVA genotype nomenclature follows that of Van Ert et al. (*4*). The new genotypes were named according to the Chinese *B. anthracis* Genetic Information Database; they are labeled “CHN” and are listed on the lines of the dendrogram. The number of anthrax cases (68) associated with the Ames lineage strains are listed in the last column

A total of 12 MLVA genotypes were identified among the 30 strains investigated here together with eight Ames lineage strains isolated in China and three strains isolated in the USA that were previously described [4] (Fig. 2 and supplementary Table S1). The three Ames strains isolated in the USA presented different MLVA genotypes than those of the Chinese strains and belonged to MLVA15–25 and MLVA15–26. Ten genotypes were identified in China; aside from the 4 genotypes (MLVA15–27, 30, 31, and 32) and 4 Chinese genotypes (CHN-1, 3, 7, and 8) reported previously, 2 new genotypes were described (according to the Chinese *B. anthracis* Genetic Information Database at the Chinese CDC). One of the new genotypes was found in Inner Mongolia, and one was found in Liaoning.

The 18 Ames lineage strains from Inner Mongolia were divided into 8 MLVA15 genotypes. While there were continual reports of outbreaks in Keyouzhongqi County, two distinctive outbreaks were caused by MLVA15–30 and MLVA15–32. Anthrax infection occurred annually in succession (2010–2015) and was caused by the MLVA15–30 genotype, which is the dominant genotype in Inner Mongolia. Moreover, two historical strains (Gansu 1954, Guangxi 1994) and four Chinese strains described previously in the literature [9] also belong to the MLVA15–30 genotype. Conversely, the strains that were isolated from Ewenkeqi, Zhalaiteqi, Linxi, Tongliao and Keyouqianqi have different genotypes.

There were 8 Ames lineage strains from Liaoning, and 6 of them isolated in two anthrax outbreaks in 2017 have identical genotype with the strains from Linxi, Inner Mongolia.

Another important genotype, MLVA15–31, was isolated from Liaoning and Xinjiang, and three previously described strains isolated in Inner Mongolia [4] belong to this genotype.

## Discussion

Human anthrax has been a continuous problem in many rural regions of China [13]. Ames lineage strains were found in the Inner Mongolia (*n* = 18), Xinjiang (*n* = 2), Liaoning (*n* = 8), Gansu (*n* = 1) and Guangxi Provinces (*n* = 1). A previous study indicated that there are 5 different lineages/groups in China and that the A.Br.Ames lineage mainly exists in Inner Mongolia [9]. In fact, the Ames lineage strains are widely distributed in northern China, including the above-mentioned provinces (Fig. 1). In previous study, the Ames lineage strains had been observed scattering from Inner Mongolia to other provinces [11], and our results provided further evidence for it. Moreover, it has been found that the epidemic caused by Ames lineage strains seems to be spreading in northeast China in recent years. The genetic diversity of these strains is illustrated by the results of the MLVA. The genetic characteristics of the Ames lineage strains from outbreaks in different provinces varied; however, strains isolated from the same outbreak grouped together. In some areas, human anthrax outbreaks occurred annually in succession, and these related strains grouped together. These observations indicate persistent *B. anthracis* spore contamination in this area. Since animal husbandry is always the major pattern of the local economy, vaccination of livestock should become the fundamental control measure.

In the USA, the Ames strain was first identified in Jim Hogg County, Texas, in 1981 and is now widely used as a reference strain [2]. Later, the Ames strain was identified as the bioterrorism agent used in the 2001 anthrax letter attacks, and at least 10 subsets of Texas isolates from animal anthrax or human anthrax cases were shown to be closely related to the Ames strain. These strains shared common ancestors that were from Inner Mongolia, China [9]. The Ames lineage was also found in Korea (*n* = 4) and Kazakhstan [14, 15]. In 2015, Derzelle reported two A.Br.Ames strains that were identified in Denmark in 1988 [16]. These reports indicate that an Ames-like isolate was probably introduced into the USA by early settlers or traders from Europe during the early to mid-1800s. The disease became established along the coastal regions of the country and then became endemic in Texas.

In a previous study, Van Ert et al. characterized 1033 *B. anthracis* isolates from 42 countries and defined their global population structure using SNPs. Three main groups and 12 subgroups were identified worldwide. These strains were divided into 221 unique MLVA15 genotypes [4]. Several other molecular genotyping methods, including SNP analysis, MLVA and single-nucleotide repeat (SNR) analysis [17–20], have also been used to illustrate the genetic relationships of *B. anthracis* strains. The canSNP results showed low resolution that was inadequate for the investigation of infectious sources. MLVA can differentiate outbreak-related strains from unrelated strains; this subtyping method allowed human anthrax cases to be linked to environmental specimens and livestock meat and products, providing information about potential infectious sources [12, 21]. MLVA can discriminate closely related strains from anthrax outbreaks [22, 23] or bioterrorism-related events [24]. From the results described in this study, different counties that lacked epidemiological links showed different MLVA patterns, indicating that the MLVA results based on 15 VNTR loci had epidemiological concordance, which is essential for epidemiological and forensic investigations. However, the diversity was observed in only 7 VNTR loci, and mainly in the highly variable pXO1 and pXO2 loci in the study. Although MLVA is the most affordable first line genotyping method in source tracing in outbreak investigation, it couldn’t provide enough information for phylogenetic analysis. With the development of next generation sequencing, more precise methods based on genome-wide SNP analysis [25, 26] will allow for the analysis of outbreak isolates and will provide insight into how mutations and microevolution shape the Ames lineage during an epizootic.

In this study, historical strains were clustered with outbreak-related strains despite large geographical distances. For example, a strain isolated from Guangxi grouped with the dominant genotype in Inner Mongolia. However, Guangxi is geographically distant from Inner Mongolia, and there is no obvious route of transmission, remote transmission may have occurred. In addition, a human anthrax outbreak occurred in Jiangsu Province in 2012, even though no historical endemic areas of anthrax were identified by national surveillance in China. By performing epidemiological investigations and molecular subtyping, the anthrax infection was found to originate in Liaoning Province in northern China, where anthrax cases are reported almost every year [12]. Highly stable *B. anthracis* spores may play an important role in the transmission of diverse genotypes via the transport and trade of contaminated commodities. So strengthening quarantine on animal and animal products is an important measure to control transmission of anthrax.

## Conclusions

The Ames lineage descended from the A.Br.001/002 lineage, which has a major presence in China. It has been also found in USA, Korea, Kazakhstan and Denmark. In the study we studied the genetic characteristics of the Ames lineage strains isolated in China by MLVA. A total of 10 MLVA genotypes were identified and 2 new genotypes were found. These strains from outbreaks in different provinces varied. The 18 Ames lineage strains isolated from Inner Mongolia were divided into eight MLVA genotypes. In some areas, human anthrax outbreaks occurred annually, and these related strains grouped together. The result showed that the MLVA method is a useful genotyping tool for *B. anthracis*, it will be helpful in the infectious sources tracing in anthrax outbreaks.

## Supplementary information

**Additional file 1: Table S1.** *B. anthracis* Ames lineage strains and the MLVA profiles identified in this study.

## Data Availability

The datasets used and/or analyzed during the current study are available from the corresponding author on reasonable request.

## Abbreviations

MLVA: Multiple-locus variable-number tandem repeat analysis
SNP: Single-nucleotide polymorphism
VNTR: Variable-number tandem repeat

## References

[1] Imperiale MJ, Casadevall A (2011). Bioterrorism: lessons learned since the anthrax mailings. MBio.

[2] Rasko DA, Worsham PL, Abshire TG, Stanley ST, Bannan JD, Wilson MR (2011). *Bacillus anthracis* comparative genome analysis in support of the Amerithrax investigation. Proc Natl Acad Sci U S A.

[3] Keim P, Gruendike JM, Klevytska AM, Schupp JM, Challacombe J, Okinaka R (2009). The genome and variation of *Bacillus anthracis*. Mol Asp Med.

[4] Van Ert MN, Easterday WR, Huynh LY, Okinaka RT, Hugh-Jones ME, Ravel J (2007). Global genetic population structure of *Bacillus anthracis*. PLoS One.

[5] Keim P, Price LB, Klevytska AM, Smith KL, Schupp JM, Okinaka R (2000). Multiple-locus variable-number tandem repeat analysis reveals genetic relationships within *Bacillus anthracis*. J Bacteriol.

[6] Ciammaruconi A, Grassi S, De Santis R, Faggioni G, Pittiglio V, D'Amelio R (2008). Fieldable genotyping of *Bacillus anthracis* and *Yersinia pestis* based on 25-loci multi locus VNTR analysis. BMC Microbiol.

[7] Lista F, Faggioni G, Valjevac S, Ciammaruconi A, Vaissaire J, le Doujet C (2006). Genotyping of *Bacillus anthracis* strains based on automated capillary 25-loci multiple locus variable-number tandem repeats analysis. BMC Microbiol.

[8] Thierry S, Tourterel C, Le Flèche P, Derzelle S, Dekhil N, Mendy C (2014). Genotyping of French *Bacillus anthracis* strains based on 31-loci multi locus VNTR analysis: epidemiology, marker evaluation, and update of the internet genotype database. PLoS One.

[9] Simonson TS, Okinaka RT, Wang B, Easterday WR, Huynh L, U'Ren JM (2009). *Bacillus anthracis* in China and its relationship to worldwide lineages. BMC Microbiol.

[10] Kenefic LJ, Pearson T, Okinaka RT, Chung WK, Max T, Trim CP (2008). Texas isolates closely related to *Bacillus anthracis* Ames. Emerg Infect Dis.

[11] Zhang H, Zhang E, He J, Li W, Wei J (2018). Genetic characteristics of *Bacillus anthracis* isolated from northwestern China from 1990 to 2016. PLoS Negl Trop Dis.

[12] Mao L, Zhang E, Wang Z, Li Y, Zhou H, Liu X (2016). Phylogenetic characteristics of Anthrax outbreaks in Liaoning Province, China, 2001-2015. PLoS One.

[13] Li Y, Yin W, Hugh-Jones M, Wang L, Mu D, Ren X (2017). Epidemiology of human Anthrax in China, 1955-2014. Emerg Infect Dis.

[14] Jung KH, Kim SH, Kim SK, Cho SY, Chai JC, Lee YS (2012). Genetic populations of *Bacillus anthracis* isolates from Korea. J Vet Sci.

[15] Aikembayev AM, Lukhnova L, Temiraliyeva G, Meka-Mechenko T, Pazylov Y, Zakaryan S (2010). Historical distribution and molecular diversity of *Bacillus anthracis*. Kazakhstan Emerg Infect Dis.

[16] Derzelle S, Girault G, Kokotovic B, Angen Ø (2015). Whole genome-sequencing and phylogenetic analysis of a historical collection of *Bacillus anthracis* strains from Danish cattle. PLoS One.

[17] Van Ert MN, Easterday WR, Simonson TS, U'Ren JM, Pearson T, Kenefic LJ (2007). Strain-specific single-nucleotide polymorphism assays for the *Bacillus anthracis* Ames strain. J Clin Microbiol.

[18] Garofolo G, Ciammaruconi A, Fasanella A, Scasciamacchia S, Adone R, Pittiglio V (2010). SNR analysis: molecular investigation of an anthrax epidemic. BMC Vet Res.

[19] Stratilo CW, Lewis CT, Bryden L, Mulvey MR, Bader D (2006). Single-nucleotide repeat analysis for subtyping *Bacillus anthracis* isolates. J Clin Microbiol.

[20] Kenefic LJ, Beaudry J, Trim C, Huynh L, Zanecki S, Matthews M (2008). A high resolution four-locus multiplex single nucleotide repeat (SNR) genotyping system in *Bacillus anthracis*. J Microbiol Methods.

[21] Liu DL, Wei JC, Chen QL, Guo XJ, Zhang EM, He L (2017). Genetic source tracking of an anthrax outbreak in Shaanxi province, China. Infect Dis Poverty.

[22] Beyer W, Bellan S, Eberle G, Ganz HH, Getz WM, Haumacher R (2012). Distribution and molecular evolution of *Bacillus anthracis* genotypes in Namibia. PLoS Negl Trop Dis.

[23] Li S, Ma Q, Chen H, Wang D, Liu Y, Wei X (2015). Genetic characterization of *Bacillus anthracis* in Guizhou Province, Southwest of China. BMC Microbiol.

[24] Hoffmaster AR, Fitzgerald CC, Ribot E, Mayer LW, Popovic T (2002). Molecular subtyping of *Bacillus anthracis* and the 2001 bioterrorism-associated anthrax outbreak, United States. Emerg Infect Dis.

[25] Girault G, Blouin Y, Vergnaud G, Derzelle S (2014). High-throughput sequencing of *Bacillus anthracis* in France: investigating genome diversity and population structure using whole-genome SNP discovery. BMC Genomics.

[26] Cummings CA, Bormann Chung CA, Fang R, Barker M, Brzoska P, Williamson PC (2010). Accurate, rapid and high-throughput detection of strain-specific polymorphisms in *Bacillus anthracis* and *Yersinia pestis* by next-generation sequencing. Investig Genet.

